# The Development and Test of a Sensor for Measurement of the Working Level of Gas–Liquid Two-Phase Flow in a Coalbed Methane Wellbore Annulus

**DOI:** 10.3390/s18020579

**Published:** 2018-02-14

**Authors:** Chuan Wu, Huafeng Ding, Lei Han

**Affiliations:** Faculty of Mechanical and Electronic Information, China University of Geosciences (Wuhan), Wuhan 430074, China; wuchuan@cug.edu.cn (C.W.); hanleicug@126.com (L.H.)

**Keywords:** working level, wellbore annulus, two-phase flow, coalbed methane

## Abstract

Coalbed methane (CBM) is one kind of clean-burning gas and has been valued as a new form of energy that will be used widely in the near future. When producing CBM, the working level within a CBM wellbore annulus needs to be monitored to dynamically adjust the gas drainage and extraction processes. However, the existing method of measuring the working level does not meet the needs of accurate adjustment, so we designed a new sensor for this purpose. The principle of our sensor is a liquid pressure formula, i.e., the sensor monitors the two-phase flow patterns and obtains the mean density of the two-phase flow according to the pattern recognition result in the first step, and then combines the pressure data of the working level to calculate the working level using the liquid pressure formula. The sensor was tested in both the lab and on site, and the tests showed that the sensor’s error was ±8% and that the sensor could function well in practical conditions and remain stable in the long term.

## 1. Introduction

China has rich CBM (coalbed methane) reserves, with 36.81 trillion m^3^ distributed underground no deeper than 2000 m, accounting for approximately 15.3% of the global reserve and ranking third in the world [1,2]. To make full use of CBM resources, the Chinese government has invested significantly in the research and development of CBM exploitation technologies in recent years, and a number of wells have been tested, aiming to obtain a complete set of technologies. However, due to limited technical conditions, vertical wells remain the major method of CBM production in the country.

In a well for CBM production, a process of drainage and depressurization is required, as fractures naturally exist in the structure of the coalbed. In this process, both the groundwater and CBM will permeate into the wellbore annulus, which results in the rise of the liquid height. On the other hand, the upward CBM bubbles and/or columns will reduce the height when they finally push out the liquid. In addition, other factors such as gushing water and leaks into the stratum will also change the height of the liquid. As a result of the above factors, the dynamic fluid level (i.e., working level) will be formed along with the continuously changing height of the liquid in the wellbore annulus.

For a vertical well, when the height of the mobile liquid level drops, the pressure around the coal reservoir also decreases. At this time, the CBM stored in the coal cracks can be released slowly to the wellbore annulus, thus continuously producing CBM. Conversely, if the working level rises, the pressure around the coal reservoir will increase. At this time, the CBM will be “pressed” in the coal crack and cannot overflow, resulting in a failure to produce coalbed methane. In addition, there is a certain functional relationship between the drop rate of the working level surface and the single well production of coalbed gas. Therefore, the working level within a wellbore annulus is a key parameter in planning the gas drainage and extraction processes, so it is necessary to make real-time measurements of the working level [3,4]. Common methods for measuring the working level include float level measurements, echo sounding, pressure detection, and mathematical modeling. These methods, however, are not applicable for the fine management of the gas drainage and extraction process for the reasons enumerated below.

### 1.1. Float Level Measurement

#### 1.1.1. Principle of the Measurement

This method was developed based on Archimedes’ principle. The float, through which a steel rope passes, is placed in the dynamic fluid with a certain length of the float below the fluid level. The float keeps its balance under the joint effect of the tensile force from the rope, the float gravity, and the buoyancy. When the height of the liquid changes, these forces on the float will also change accordingly. Thus, the working level can be calculated by detecting the forces on the float and by measuring the length of the steel rope [5].

#### 1.1.2. Disadvantages

- This method requires a smooth wall of the well (i.e., equipped with a casing); otherwise, the float will be scraped or even entrapped by the irregular wall, which will cause measurement errors.
- This method imposes higher requirements on the fabrication of the float. If the float is too light, the foam layer in the wellbore annulus may not only block the float when put to the working level but also generate interference with the forces on the float. If the float is too heavy, it will have a larger size, which renders it easily trapped in the relatively small CBM wellbore annulus (generally 26 mm), which will cause large measurement errors. For the fabrication of the float, the liquid density must be taken into account. However, in a CBM wellbore, the density of the two-phase flow changes with the gas content, so it is impossible to make an appropriate float based on density.
- This method requires the open wellbore. To prevent CBM leakage, however, the mouth of the wellbore must be sealed with a device.

From the above, we can see that this method cannot make fine management and real-time measurements of the dynamic CBM fluid level.

### 1.2. Echo Sounding Measurement

#### 1.2.1. Principle of the Measurement 

An acoustic generator mounted on the wellhead is used to generate a beam of acoustic waves. During its movement downward to the bottom of the wellbore, the acoustic wave will reflect when it meets the sound mark, tubing coupling, and fluid level. The reflected wave will then return to the wellhead and be recorded by a rheomicrophone. The working level can be calculated by measuring the acoustic velocity and the reflection interval [6,7,8].

#### 1.2.2. Disadvantages

- The acoustic attenuation will result in large measurement errors due to the relatively small size of the CMB wellbore annulus (generally 26 mm).
- On the working level in the CMB wellbore annulus, there is a long foam section with a length of tens or even a hundred meters and can adsorb a great amount of acoustic energy and therefore cause large measurement errors.
- During CBM production, the friction between the sucker rod and the tube will generate an interference wave that has a significant effect on measurement.
- Typically, an echo device is equipped with a sound gun to generate the acoustic wave, but fire is strictly prohibited on oil and other gas production sites, so this method carries a potential safety threat.
- Acoustic propagation is weak when the casing pressure is negative, which invalidates the method.
- As described above, this method cannot achieve fine management and real-time dynamic CBM level measurement.

### 1.3. Pressure Detection Measurement

#### 1.3.1. Principle of the Measurement 

**Figure 1:**
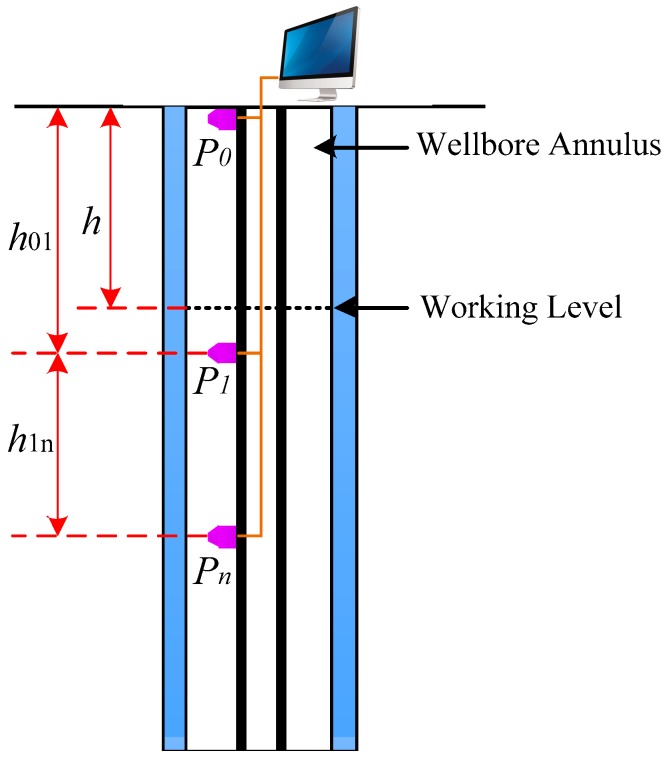
Principle of the pressure detection measurement.

Figure 1 shows the basic principle of the pressure detection measurement. A number of pressure sensors are placed at different positions in a CBM wellbore annulus, and the values read from the sensors are represented by *P*_0_, *P*_1_, …, *P_n_*, respectively. *P*_0_ is the pressure at the wellhead, and *P*_1_ is the pressure at a position very near to the working level. Providing that the depths of the sensors are known [9,10,11], then

(1) $$\rho_{1}=\left(P_{n}-P_{1}\right)/gh_{1n}$$

where *ρ*_1_ is the mean density of the *P_n_P*_1_ section; *h*_1*n*_ is the length of the *P_n_P*_1_ section (a known quantity); *g* is a constant.

Thus, the working level can be expressed as

(2) $$h=h_{01}-\left(P_{1}-P_{0}\right)/\rho_{1}g$$

where *h* is the working level; *h*_01_ is the height from *P*_1_ to the wellhead (a known quantity).

#### 1.3.2. Disadvantages

In this method, the density near the working level is replaced by the density at a certain position below the working level. Hence, this method is applicable to a well where the density changes slightly. However, for a CBM well (especially when there is more than one coalbed), the methane moves continuously from the coalbed into the wellbore annulus, which results in a large variation of the densities at different depths and therefore causes large measurement errors.

According to the above, it is clear that this method cannot achieve fine management and real-time dynamic CBM liquid level measurements.

### 1.4. Mathematical Modeling Calculation

#### 1.4.1. Principle of the Measurement

Generally, this method does not require any detection devices. It relies on the existing apparatus on the well field and relevant data to calculate the working level by using a mathematical model. Common mathematical modeling measurements include the indicator diagram method and the pressure-out method. 

The indicator diagram method consists in using the sucker rod system’s parameters to calculate the working level. The working level has a complicated functional relationship with the parameters of the sucker rod system such as the suction pressure, the saturation pressure, temperature, length of stroke, and so on. Therefore, the working level can be calculated by deriving the functional relation [12]. The pressure-out method is a method that uses the pressure-out of the pump to calculate the working level. The pressure-out of the pump has a functional relationship with known parameters, such as displacement, the head and power of the pump, and the speed, and unknown parameters, such as the frictional resistance loss, the initial pressure of the pump when pumping, and the working level. Therefore, the working level can be calculated by deriving the functional relation [13].

#### 1.4.2. Disadvantages

The error of this method depends largely on the model as well as on relevant parameters. Theoretically, high accuracy can be reached if the model is well built and if the parameters are selected carefully. According to the different types of parameters, the error of the indicator diagram method is between 1.69% and 23.3% [12,14], while the error of the pressure-out method varies between 3% and 15% [13]. For this mathematical model, however, both the calculation and the follow-up analysis can be very complicated. In addition, it cannot make real-time measurements of the working level.

It is clear that this method cannot achieve fine management and real-time measurements of the dynamic CBM fluid level.

From the analysis above, it is clear that the four types of methods summarized are the existing methods for measuring the working level for CBM, but the applications of the existing methods are limited by the environmental conditions and all carry different shortcomings. For example, the errors of the buoy method and echo sounding are too large. Echo sounding is more suitable for the wellbore without foam layer, while the pressure-out method is more suitable for a single coal seam, the error of which is very large when measuring multiple coal seams. The mathematical modeling method requires complex calculations and measurements where errors related to the model and parameter type cannot achieve real-time measurement. Moreover, these methods also have problems relating to installation, part design, safety, and error and as a result are not applicable for real-time measurement of the working level in a CBM wellbore annulus. For this reason, we designed a new sensor for measuring the working level. This sensor can make real-time and accurate measurements of the working level and is more suitable for the environment described above, with fewer errors than the existing measurement methods measuring the foam layer wells and multiple coal seams.

## 2. Measurement Indicators and Basic Principles

The index of the sensor must be confirmed before designing the sensor, after which the overall scheme can be designed according to the measurement index.

### 2.1. Measurement Indicators

The analysis of the measurement index is summarized as follows and is based on the real working environment of CBM wellbores.

#### 2.1.1. Sealability

The designed sensor was installed under the working level so that we could assume that the maximum depth of the designed sensor was 400 m below the working level and the water pressure was 4 MPa according to the liquid pressure formula. Thus, the designed sensor must be able to bear a water pressure of 4 MPa.

#### 2.1.2. Temperature

According to the geothermal gradient, the temperature will increase 3 °C per 100 m from the Earth’s surface [15], and the temperature will increase by 36 °C when assuming that the maximum depth of designed sensor is 1200 m under the Earth’s surface according to the actual condition of the CBM well. Thus, the designed sensor’s temperature range was designed to be 0–85 °C.

#### 2.1.3. Measurement Range

The measurement range of the designed sensor was 0–1200 m according to the numerous investigations [16,17,18,19,20] on CBM wells (including references and field visits) and the average range of the CBM well.

#### 2.1.4. Error

According to the needs of the real project and the actual situation of achievable precision, the error of the designed sensor was ±8%.

To sum up, the designed sensor’s measurement index is shown in Table 1. 

### 2.2. Basic Principle

**Figure 2:**
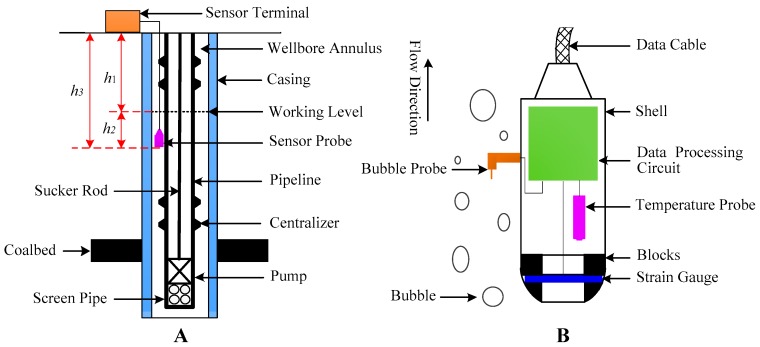
Basic principle of the sensor measurement. (**A**): the installation instruction of the sensor; (**B**): the basic structure of the sensor.

Figure 2 shows the basic principle of the sensor measurement, and Figure 2A is a schematic map of the structure of a CBM extraction wellbore. As shown in Figure 2A, the sensor consists of a probe and a terminal. Before starting the sensor, the known and necessary parameters must be input manually into the terminal. Then, the probe begins to conduct the real-time collection of well data that are simultaneously transferred to the terminal where the data will be processed and used to calculate the working level. Specifically, the working procedure of the sensor is as follows:

① Mount the probe under the working level; the depth *h*_3_ is a known quantity. Before the sensor is lowered into the wellbore, it is necessary to obtain the fluid level at this stage. Only according to the current data can we obtain *h*_3_.
② Power on the terminal and manually input the value of *h*_3_ and the wellhead pressure *P*_0_ into the terminal.
③ Power on the probe. The probe will collect data on the pressure at the mounting position (*P*_1_) and the mean density of the *h*_2_ section (*ρ*) in real time. The *P*_1_ and *ρ* data will then be simultaneously transferred to the terminal.
④ The data are processed in the terminal to calculate the working level (*h*_1_), which will also be displayed on and stored in the terminal. The calculation at the terminal is as follows:

A. Calculate the pressure (*P*_2_) of the water at the position where the sensor probe is mounted:

(3) $$P_{2}=P_{1}-P_{0}$$

where *P*_1_ is the pressure measured by the sensor and *P*_0_ is the wellhead pressure.

B. Calculate the value of *h*_2_ according to the liquid pressure formula:

(4) $$h_{2}=P_{2}/\rho g$$

where *h*_2_ is the distance from the probe to the working level as shown in Figure 2A; *ρ* is the mean density of the liquid corresponding to section *h*_2_; and *g* is the gravity constant.

C. Calculate the working level (*h*_1_):

(5) $$h_{1}=h_{3}-h_{2}$$

where *h*_3_ is the distance from the probe to the wellhead as shown in Figure 2A.

As suggested by the description above, the basic function of the sensor probe is to measure the pressure *P*_1_ at the mounting position and the mean density *ρ* corresponding to section *h*_2_. Figure 2B shows the basic structure of the accordingly designed probe. When the two-phase flow in the wellbore annulus flows along the direction of the arrow (the flow is in fact the upward movement of the bubbles in the two-phase flow), the operation of the sensor probe can be divided into three steps: 

First, measure the pressure *P*_1_ at the position where the probe is mounted.

Second, measure the liquid density *ρ*_1_ also at the probe mounting site; *ρ*_1_ is used to replace the mean density ρ of the liquid corresponding to section *h*_2_.

Finally, transfer *P*_1_ and *ρ* simultaneously to the terminal via a cable.

During the above procedure, the pressure *P*_1_ and the densities *ρ* are measured and calculated according to the following procedure:

(1) Measurement of the Pressure *P*_1_

As shown in Figure 2B, a strain gauge is placed in the chamber of the housing and is fixed by blocks to seal the inner wall of the housing. The strain gauge will become deformed under the pressure from the two-phase flow in the wellbore annulus, and there is a linear relationship between the pressure and the deformation; i.e., the greater the pressure, the greater the deformation. Therefore, the pressure can be acquired by calibrating the deformation of the strain gauge. However, the strain gauge is sensitive to temperature, and this sensitivity can easily generate temperature drift. Hence, a temperature probe needs to perform real-time measurements of temperature in order to compensate for drift. The data of both the strain gauge and the temperature probe are transferred to a processing circuit in real time where the data will be processed and calculated.

(2) Calculation of the Fluid Density *ρ*

Inside a CBM wellbore annulus is a gas–liquid two-phase flow, which, if measured with an ordinary density sensor, will always show the density of the liquid only. The density of the gas–liquid two-phase flow, however, is the mean density of the mixture of the gas and the liquid, which cannot be measured using an ordinary density sensor. According to the content of the gas, the gas–liquid two-phase flow can be divided into different patterns that correspond to different densities. For this reason, we designed a “bubble probe” and used it to measure the fluid data in real time and transmitted that data to a processing circuit. There, the pattern of the fluid is identified by calculation, whereby the density of the fluid can be determined according to the pattern of the fluid.

## 3. Design of the Sensor

From the description of the sensor principle above, we know that a technical difficulty in designing the sensor is reflected in two aspects: the pressure measurement (named the pressure measurement module) and the fluid density measurement (named the fluid density measurement module). Below, the details of the two modules are provided.

### 3.1. Pressure Measurement Module

As shown in Figure 2B, the fluid pressure is acquired by measuring the deformation of the strain gauge, which will certainly generate temperature drift and null drift. Therefore, both a hardware circuit and software fitting are required to solve the two drifts.

#### 3.1.1. Null Drift

**Figure 3:**
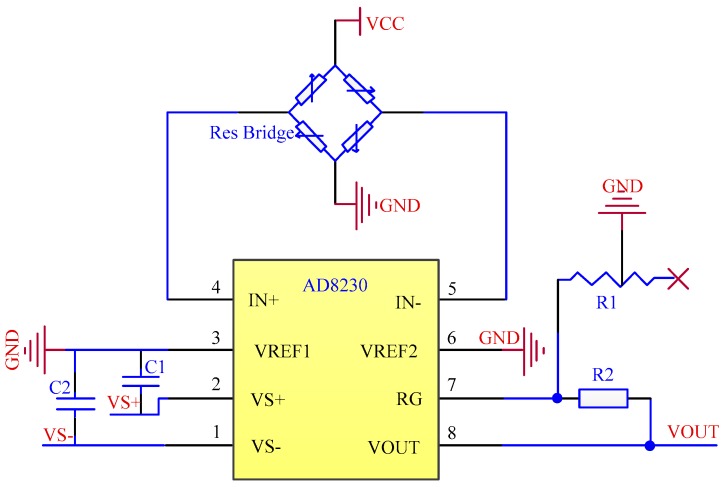
Circuit diagram of null drift processing.

The original output data of the sensor should be 0 when the load is 0 and the temperature is constant, but the output data are not 0, i.e., null drift occurs. Causes of null drift include initial deformation of the strain gauge and the irreversible and permanent residue deformation of the strain gauge when it suffers a large hit. The AD8230 chip (Analog Devices, Norwood, MA, USA) was used for building the hardware circuit to solve the problem of null drift, as shown in Figure 3 [21]. 

#### 3.1.2. Temperature Drift

**Figure 4:**
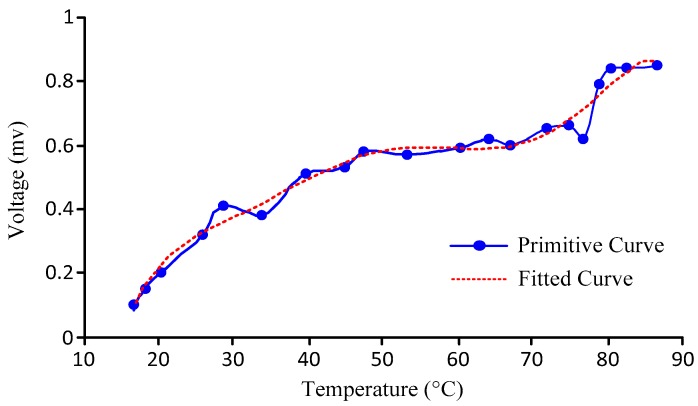
Calibration fitting curve of temperature drift.

Given no load and no null drift, the output data of the designed sensor should theoretically be 0; however, in fact, it is not. Temperature drift occurs. Known causes of temperature drift include differences in the resistances of the strain gauges and the inhomogeneity of the material in which the strain gauge is pasted when heated [21]. The method of software compensation was used to solve the temperature drift, as enumerated below:

① Place the sensor installed with the pressure measurement module in a device whose temperature can be regulated and ensure no load when testing. We performed the test with a water bath.
② Turn on the device (i.e., water bath) and adjust the holding temperature of the device. Then, start the pressure measurement module when the temperature is stable. Record both the temperature and output signal of the pressure measurement module.
③ Continue adjusting the holding temperature of the device and repeat Step ②.
④ Power off the device and the pressure measurement module.
⑤ By fitting the obtained data, we can find the functional expression between the temperature and the output signal. The fitting curve of the sensor designed in this paper is shown in Figure 4, and the functional expression can be obtained as shown in Equation (6) according to the data shown in Figure 4:
  (6) $$y=-4e^{-10}x^{6}+e^{-7}x^{5}-2e^{-5}x^{4}+0.001x^{3}-0.0338x^{2}+0.5858x-3.8948$$
  where *y* is the voltage value of the output signal, and *x* is the temperature.

The curve fitting formula is not representative, and the formula is listed in this paper only to illustrate the method of addressing temperature drift and how to fit the curve. Using a different brand of strain gauge in the production of the sensor, the material, and the texture and even using an uneven amount of glue will lead to a different temperature drift, and the fitting formula will be different. Therefore, in the production of the sensor, the method proposed here must be followed to fit the corresponding formula. 

⑥ Program the equation obtained in Step ⑤ into the data processing chip of the sensor.

### 3.2. The Fluid Density Measurement Module

The two-phase flow of the wellbore annulus can be divided into different patterns according to the gas content, and the different patterns correspond to different densities of the two-phase flow. Therefore, we can automate the identification of the two-phase patterns by using the bubble probe, which was designed by the author of this paper, and then obtain the fluid density by calibrating the functional expression between the patterns and the fluid density according to repeated experiments.

#### 3.2.1. The Introduction of Detection Principles of the Bubble Probe 

**Figure 5:**
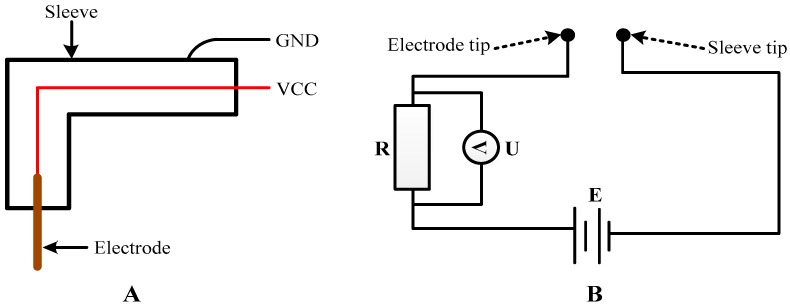
Detection principle of the bubble probe. (**A**): the elementary structure of the bubble probe; (**B**): the data process circuit of the bubble probe.

Figure 5 shows the basic detection principle of the bubble probe. Figure 5A shows the elementary structure of the bubble probe, and Figure 5B shows the data process circuit of the bubble probe [22]. As shown in Figure 5A, the bubble probe consists of a sleeve and an electrode. The sleeve connects with the VCC (the positive pole of the power), while the electrode connects with the GND (the negative pole of the power). The test device of the conductivity is built when the power is open [22]. 

The conductivity of the gas is 0, while the liquid is not 0, so we can capture the bubbles according to the apparent difference in conductivity between the two media. Figure 5B is used to detect the bubbles where *E* is the voltage of the power supply, *R* is the resistance, and *U* is the voltage between the two ends of the *R*. When the medium connecting the sleeve and the electrode is liquid, the circuit shown in Figure 5B is turned on, as the liquid conductivity is not 0. Thus, the *U* is not 0. Conversely, when the medium is gas connecting the sleeve with the electrode, the circuit shown in Figure 5B is turned off, as the gas conductivity is 0; hence, the *U* is 0. We can therefore capture the bubbles by judging the value of *U*. 

#### 3.2.2. Automatic Identification Principles of the Two-Phase Flow

The automatic identification of the two-phase flow is related to the patterns of two-phase and its graph, so we introduce the patterns of the two-phase flow and its graph first and then introduce the automatic identification principles.

(1) The Patterns of the Two-Phase Flow and Its Graph

**Figure 6:**
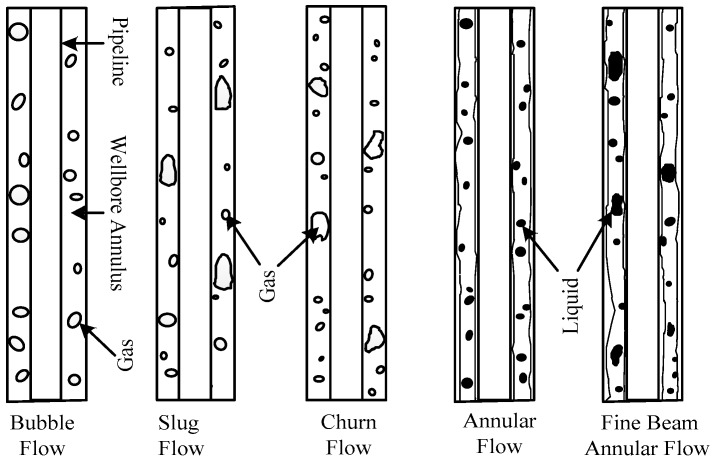
Pattern graph of two-phase flows in vertical ascending pipelines.

The gas–liquid two-phase flow can be divided into different patterns according to the angle of the pipeline where the two-phase flow is located. As the research regarding CBM wells consists in vertical ascending pipelines, the two-phase flow in a CBM well can be analyzed in terms of bubble flow, slug flow, churn flow, annular flow, and fine beam annular flow, which change as gas content increases [22], as shown in Figure 6.

The graph of every pattern can be obtained by using the bubble probe (shown in Figure 5) to monitor the two-phase flow (shown in Figure 6). The graph of a two-phase flow is the basis of the two-phase flow’s automatic identification.

**Figure 7:**
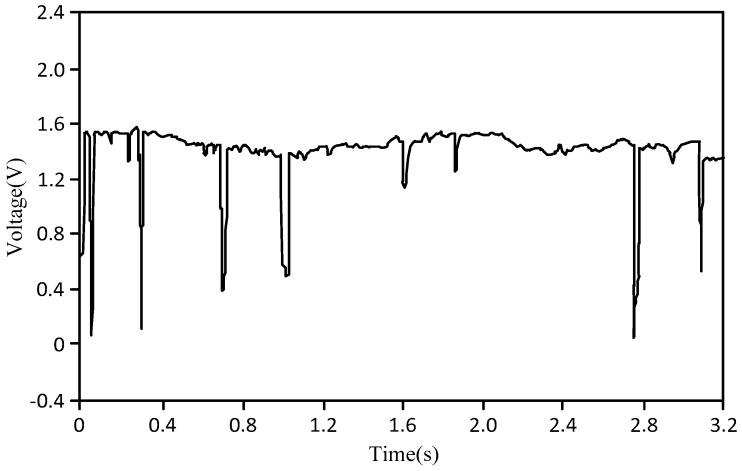
Graph of bubble flow.

Figure 7 shows the graph of the bubble flow where the abscissa is the test time (unit: s), and the ordinate is the output signal voltage value of the bubble probe (unit: V). As shown in Figure 7, the key characteristic of a bubble flow is that there are many bubbles at one time.

**Figure 8:**
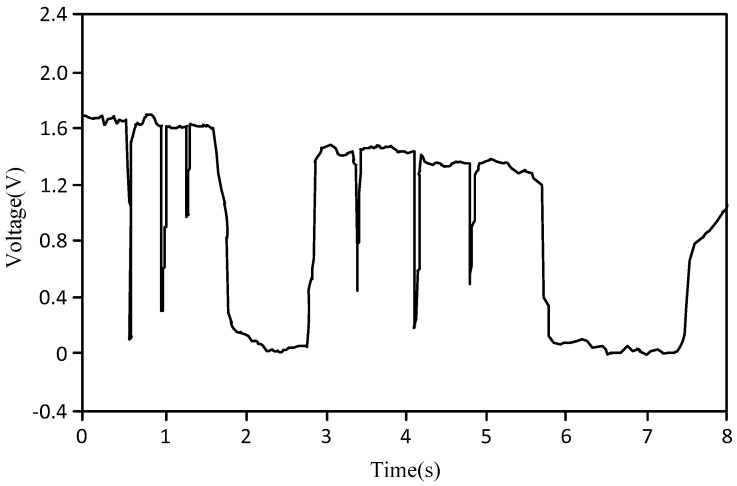
Graph of slug flow or churn flow.

Figure 8 is the graph of the slug flow or churn flow, where the abscissa is the test time (unit: s), and the ordinate is the output signal voltage value of the bubble probe (unit: V). As shown in Figure 8, the key characteristic of slug flow or churn flow is that there is only one bubble at a time, and the liquid phase occurs at a different time. As the slug flow and churn flow have similar characteristics, they are grouped together.

**Figure 9:**
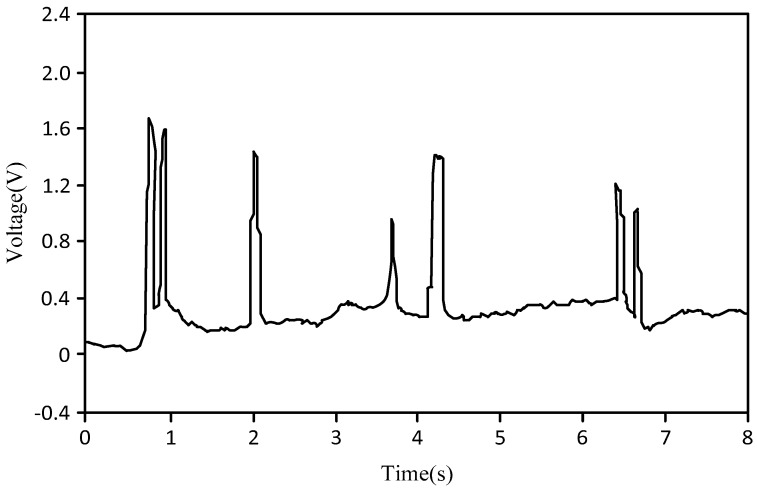
Graph of annular flow or fine-beam annular flow.

Figure 9 shows the graph of the annular flow or fine-beam annular flow, where the abscissa is the test time (unit: s), and the ordinate is the output signal voltage value of the bubble probe (unit: V). As shown in Figure 9, the key characteristic of the annular flow or fine-beam annular flow is that a single bubble mixed with a short-term liquid phase occupies the entire certain time. As the annular flow and the fine beam annular flow have similar characteristics, they are grouped together.

(2) Introduction to the Automatic Identification Principles of the Two-Phase Flow

From Figure 6, it is clear that the basis of the division of two-phase flow patterns is gas content, i.e., as the gas content increases, the pattern of the two-phase flow gradually changes from bubble flow to fine beam annular flow. The appearance of this process is that the diameter of the bubble gradually increases and the bubbles finally link together from the bubble flow to the fine beam annular flow. Thus, we can obtain the basic characteristics of the two-phase flow from Figure 7 to Figure 9 and obtain the automatic identification principles of the two-phase flow further as shown in Table 2 [21]. The details are as follows:

① If multiple bubbles are detected by the bubble probe during time 0–*t*_1_, then it is recognized as the bubble flow.
② If one bubble occupies the entire time of 0–*t*_1_ and the liquid phase occurs from time *t*_1_–*t*_2_, then it is recognized as slug flow or churn flow. As slug flow and churn flow have similar characteristics, they are grouped together.
③ If one bubble mixed with a brief liquid phase occupies the entire time of 0–*t*_3_, then it is recognized as annular flow or fine beam annular flow. As the annular flow and the fine beam annular flow have similar characteristics, they are grouped together.
④ If the gas phase occupies the entire time of 0–*t*_3_, then it is recognized as the gas phase.
⑤ If the liquid phase occupies the entire time of 0–*t*_3_, then it is recognized as the liquid phase.

Thus, the automatic identification principles of the two-phase flow can be summarized as follows:

First, the data of the bubble probe is read via the sensor’s data processing chip to determine whether bubbles are present and calculate the time taken for each bubble to pass through.

Next, the automatic discrimination of the flow pattern is performed according to the rules described in Table 2 and Rules ①–⑤.

The values of *t*_1_, *t*_2_, and *t*_3_ (shown in Table 2) are crucial to the automatic identification of two-phase flow patterns, and a large error will be produced if *t*_1_, *t*_2_, and *t*_3_ have improper values. The values of *t*_1_, *t*_2_, and *t*_3_ are related to the size of the bubble probe, the sample frequency, the data processing rate of the microprocessor, and so on, so a single test will not return accurate calculations. The only way to obtain the values of *t*_1_, *t*_2_, and *t*_3_ is through repeated testing. The test equipment and test steps are shown below.

(1) Test Equipment

**Figure 10:**
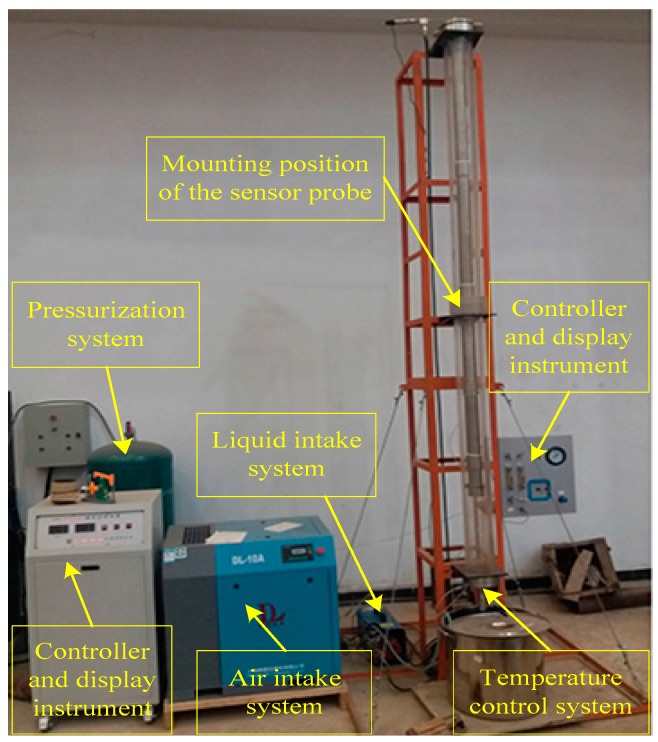
The two-phase flow simulator.

The test equipment is performed on a purchased two-phase flow simulation device (hereinafter referred to as the simulator), as shown in Figure 10. This simulator can simulate all patterns of the two-phase flow in a CBM wellbore annulus. It can also simulate the pressure, temperature, and the flow in the wellbore. In addition, the simulator has a data collection function allowing it to make real-time measurements of the parameters of the simulated two-phase flow (including the working level) and display the parameters. The detailed parameters of the simulator are as follows.

(2) Test steps

① The bubble probe of the developed sensor is mounted on a two-phase flow simulator.
② By adjusting the amount of air intake of the simulation device, the flow pattern is simulated, and the flow pattern results of the bubble probe are recorded.
③ Each flow pattern is subjected to 10 tests, and the results obtained from the bubble probe are compared with the real results, allowing us to find the flow pattern discrimination error.
④ Change the *t*_1_, *t*_2_, and *t*_3_ values in the probe parameters and repeat the experiment in Steps ①–③; then, the flow pattern discrimination error is obtained again.
⑤ The values of *t*_1_, *t*_2_, and *t*_3_ obtained when the error is minimized are taken as the final value.

The values of the sensor designed in this article are such that *t*_1_ = 2.6 s, *t*_2_ = 3.9 s, and *t*_3_ = 4.5 s.

Height of the wellhole: 4 m.Size of the fixed base: 1.5 m × 1.5 m.Casing diameter: 124.26 mm.Tubing diameter: 73 mm.Liquid flow range: 0–162 L/min.Gas flow range: 0–1.42 m^3^/min.Liquid flow direction: from top to bottom. Gas flow direction: from bottom to top.Pressurization range: 0–12 MPa.Temperature working range: from ambient temperature to 100 °C.

#### 3.2.3. Calibration of Pattern-Density

The functional relationship between the pattern and density of the two-phase flow was calibrated on the basis of a great number of experiments. Detailed information about the calibration test is provided below as follows:

(1) Calibration Apparatus
  The calibration test is performed on the simulator as shown in Figure 10.
(2) Calibration Steps
  ① Mount the pressure sensor on the transparent wellbore of the simulator to measure the pressure of the liquid column.
  ② Pour water into the transparent wellbore. Ensure that the pressure sensor is below the liquid and the pressure inside the wellbore annulus is equal to the standard atmospheric pressure.
  ③ Turn on the simulator and the pressure sensor.
  ④ Adjust the air inflow of the simulator to ensure that the pattern of the two-phase flow is a bubble flow. As different flow patterns have their own obvious characteristics and the characteristic of each flow pattern is obvious, they can be identified with the naked eye.
  ⑤ Read the working level shown on the simulator; record both the pressure measured by the pressure sensor, and the pattern of the two-phase flow.
  ⑥ Substitute the data obtained in Step ⑤ into the liquid pressure formula to calculate the mean density of the fluid.
  ⑦ Gradually change the air inflow of the simulator to simulate all fluid patterns and repeat Steps ⑤ and ⑥.
  ⑧ Power off the entire system after the calibration test is finished.
(3) Calibration Results

The relationship between the pattern and the density of the two-phase flow was calibrated on the basis of a great number of experiments (we carried out 50 groups of experiments for each type of flow). This relationship was programmed into the sensor to realize the function that measures the fluid density using a bubble probe.

Table 3 shows the relationship between the patterns and the densities of the two-phase flow. Again, as shown in Figure 6 and Table 2, where slug flow and churn flow were calibrated as one pattern since they had similar features; for the same reason, annular flow and fine beam annular flow were also calibrated as one pattern.

## 4. Testing

Tests were performed both in the lab and on site. The tests performed in the lab examined the measurement error of the sensor, while the on-site testing checked the stability of the sensor under practical conditions over a long period.

### 4.1. Test in the Lab

The sensor was tested in the lab to analyze the measurement errors including the overall error, the error of measuring each fluid pattern, the effect of temperature on measurement, and the effect of pressure on measurement.

**Figure 11:**
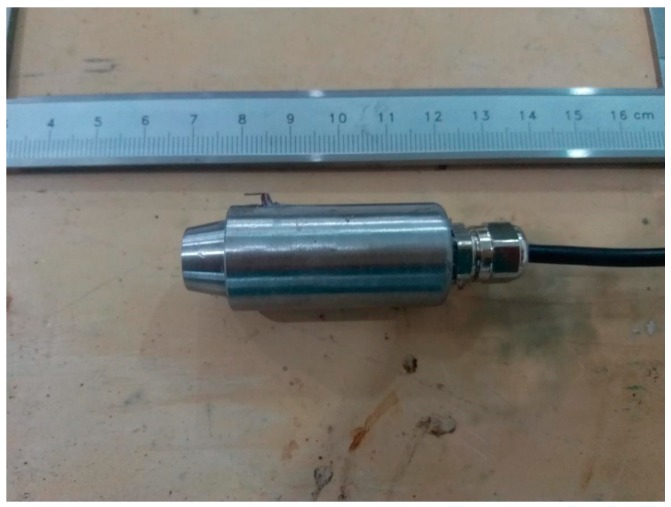
A picture of the finished sensor probe.

Figure 11 shows the finished sensor probe. The designed sensor was tested with the simulator, as shown in Figure 10. The test procedure is as follows:

① Mount the sensor probe at the transparent wellbore of the simulator.
② Pour water into the transparent wellbore. Ensure that the sensor probe is below the liquid.
③ Turn on the simulator and the sensor.
④ Adjust the air inflow of the simulator to ensure that the pattern of the two-phase flow is bubble flow.
⑤ Adjust the pressure and the temperature in the wellbore annulus of the simulator to ensure that the environment where the sensor probe is positioned is the same as the real wellbore environment.
⑥ Manually input the values of the depth of the sensor probe and the pressure in the wellbore annulus into the terminal of the sensor.
⑦ Read the working level shown on the simulator and call it the actual height. Record the data output from the sensor at the same time and call it the measured height.
⑧ Change the air inflow of the simulator to simulate all fluid patterns.
⑨ Repeat Steps ⑤–⑧
⑩ Turn off the simulator and the sensor after the test is over.

The statistical analysis of the test data of the 200 groups of experiments showed that the overall measurement error was smaller than 8%, and 75% of the test data errors were lower than 6%. The error of the float level measurement was related to the thickness of the foam layer, the fluid density, the craftsmanship of the float, and so on. Thus, the error of this method was very large and was eliminated. The error of the echo sounding measurement was nearly 33% when the foam layer was thicker [9,23], the error of the pressure detection measurement was lower than 13% [23,24,25], and the error of the mathematical modeling calculation was between 1.69% and 23.3% [12,13,14]. Thus, the measurement error of our sensor was higher than that of conventional measurement tools.

**Figure 12:**
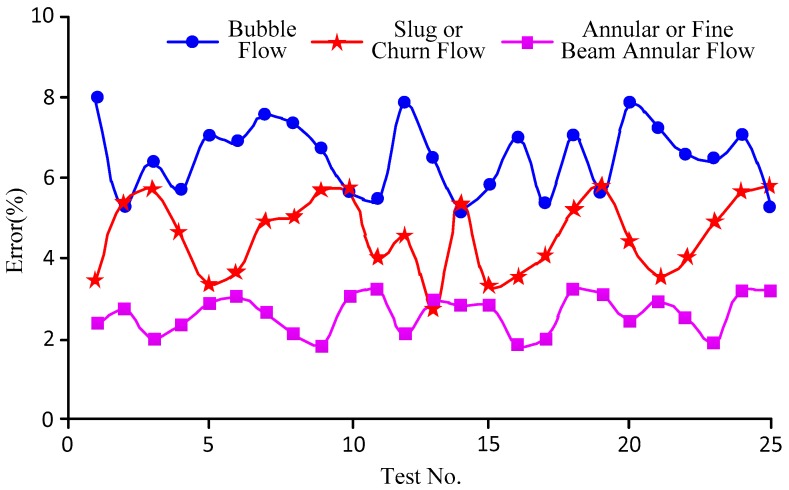
Error curves corresponding to different patterns.

By carrying out the classification and statistical analysis of the test data in accordance with different fluid patterns, we obtained the error curves as shown in Figure 12 where the abscissa represents the test number and the ordinate represents the error. The following conclusions can be drawn from Figure 12:

① The majority error of the sensor was 5.2–8% for measuring bubble flow, 2.6–5.9% for measuring slug flow or churn flow, and 1.8–3.2% for measuring annular flow or fine beam annular flow.
② The error of the sensor measuring bubble flow was the highest and was the lowest when measuring the annular flow or fine beam annular flow. This was because the basic principle of the sensor was the measurement of the densities of different fluid patterns, among which the density of the bubble flow could vary in a wide range. The sensor, however, was designed to measure the mean density of the bubble flow, which was the cause of the large error. For the annular or fine beam annular flow, the density range was narrow, so the error was relatively small. Likewise, the density range of the slug or churn flow was between the density ranges of the above two patterns.
③ When measuring the slug or churn flow, the errors of the sensor fluctuated greatly: some were higher than the errors in measuring the bubble flow and some were lower than the errors in measuring the annular or fine beam annular flow. This was because according to the gas content, the two-phase flow could be classified into bubble flow, slug flow, churn flow, annular flow, and fine beam annular flow; however, the transition from bubble flow to slug flow was wide and not explicit, i.e., at a certain gas content, the pattern could either be bubble flow or slug flow. Likewise, the transition from churn flow to annular flow was not explicit but narrow, which was relatively better than the bubble-to-slug transition.

**Figure 13:**
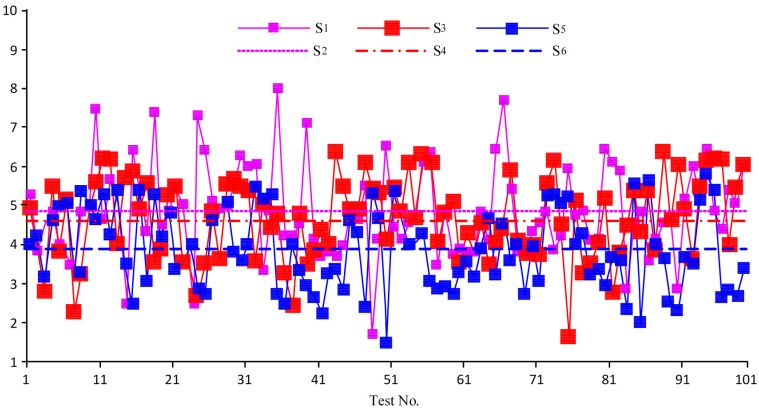
Measurement error curves of the sensor when measuring the bubble flow under different temperatures. S_1_: error at 16 °C and under standard atmospheric pressure; S_2_: mean value of S_1_; S_3_: error at 58 °C and under standard atmospheric pressure; S_4_: mean value of S_3_; S_5_: error at 90 °C and under standard atmospheric pressure; S_6_: mean value of S_5_.

Figure 13 shows the errors of the sensor in measuring the bubble flow pattern when only changing the temperature of the fluid while keeping other measurement conditions including the standard atmospheric pressure in the wellbore annulus, unchanged. As all the impacts of temperature on bubbles of different flow patterns were the same, i.e., the bubble volume of thermal expansion and contraction, the text does not list the other flow patterns’ data. 

As shown in Figure 13, the errors under different temperatures changed randomly and irregularly. There was a certain regularity in mean values: the mean values gradually decreased as the temperature increased, namely, S_6_ < S_4_ < S_2_.

The cause of this phenomenon was related to the error of the bubble probe. As shown in the ideal gas state equation (Equation (7)), when the temperature of the two-phase flow changed, the volume of the bubbles in the fluid also changed, and the volume was larger if the temperature was higher. The error increased because it was difficult for the probe to capture small bubbles. Bubble volume will increase at a higher temperature, and this decreases the error of the bubble probe. This is the reason why the measurement error is slightly higher at a lower temperature.

(7) PV=nRT

where *P* is the environmental pressure; *V* is the gas volume; *n* is the quantity of the gas; *R* is the ideal gas constant; *T* is the thermodynamic temperature of the ideal gas.

The values of S_6_, S_4_, and S_2_ were different due to the thermal expansion and contraction of the bubble. The bubble volume in the thermal expansion and contraction of the change will not be particularly large: therefore, the impact is limited. Hence, the S_6_, S_4_, and S_2_ values were not equal, but were relatively close. 

**Figure 14:**
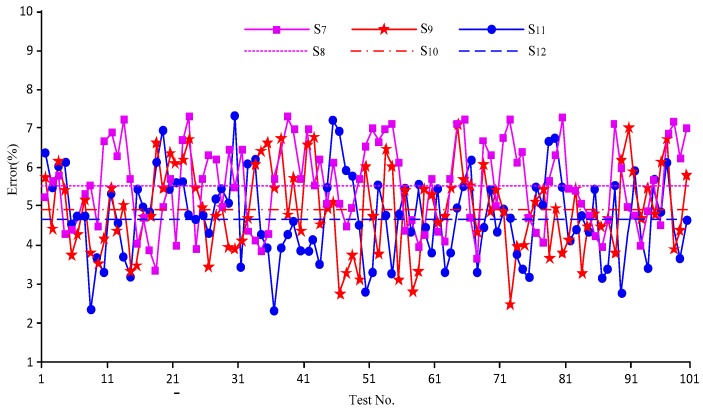
Measurement error curves of the sensor when measuring the bubble flow under different pressures. S_7_: error under the pressure of 0.8 MPa and at 20 °C; S_8_: mean value of S_7_; S_9_: error under 0.4 MPa and at 20 °C; S_10_: mean value of S_9_; S_11_: error at 0.1 MPa and at 20 °C; S_12_: mean value of S_11_.

Figure 14 shows the errors of the sensor in measuring the bubble flow pattern by only changing the pressure in the wellbore annulus while keeping the other measurement conditions, including the temperature of 20 °C, unchanged. As all the impacts of pressure on the bubbles of different flow patterns were the same, i.e., the volume deformation of the bubbles under different pressures, the text does not list the data of the other flow patterns. 

As shown in Figure 14, the errors under different pressure changed randomly and irregularly, but there was a certain regularity in the mean values: the mean values gradually decreased as the pressure decreased, namely, S_12_ < S_10_ < S_8_.

The cause of this phenomenon was related to the error of the bubble probe. As also known from Equation (7), when the pressure in the wellbore annulus changed, the volume of the bubbles in the fluid also changed, and the volume was lower if the pressure was higher. The error increased because it was difficult for the probe to capture small bubbles. The bubble volume will shrink under a higher pressure, which slightly increases the error of the bubble probe. This is the reason the sensor error was slightly larger under a higher pressure.

The values of S_8_, S_10_, and S_12_ were different due to the volume deformation of the bubbles under different pressures. The impact was limited, however, as the test pressure was not high, and the values of S_8_, S_10_, and S_12_ were not equal, but relatively close.

### 4.2. Test on Site

The on-site test evaluated the stability of the sensor under practical conditions over a long period. The test procedures are as follows.

**Figure 15:**
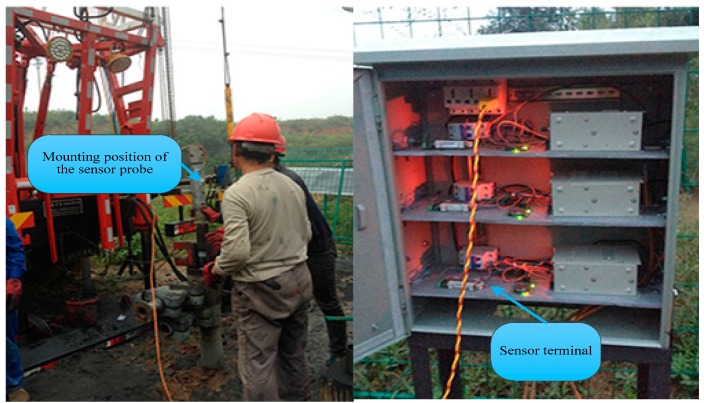
Test on site.

Test Procedure: Mount the sensor probe on a custom-designed gauging nipple that is connected to the pipeline (as shown in Figure 15). Control the depth of the pipeline to place the sensor probe at the designated depth of the CBM well and then turn on the power. The data collected by the sensor probe will be simultaneously transferred to, displayed on, and stored in the sensor terminal via a cable. The sampling frequency of the sensor probe is 200 Hz, and the memory capacity of the sensor terminal is 8 GB. The overall power consumption is 103 ma and the expected lifetime of the system is 8 months.

Test Location: Well JS-064, Lanyan CBM Cooperation, Jincheng City, Shanxi Province, China.

Test Period: From 11 September 2015 to 28 June 2016, during which the operations of the sensor were stopped intermittently for routine maintenance as well as to clear memory of the sensor. The experiment lasted more than 9 months, which exceeded the expected lifetime of the system.

**Figure 16:**
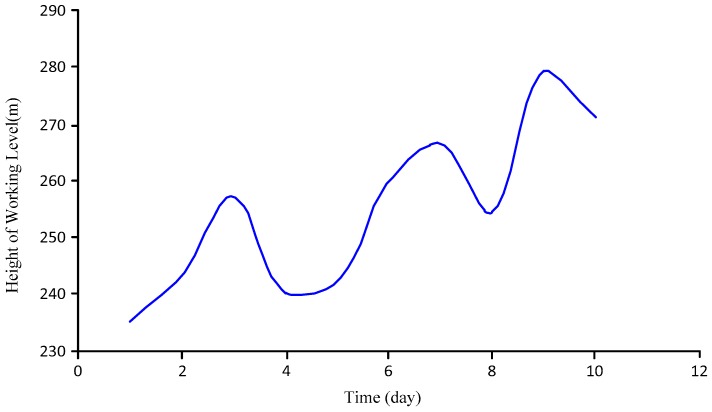
On-site data during a continuous period.

Test Conclusions: Figure 16 shows the test data during a continuous period. As shown, the working level fluctuated upwards. By looking up the site’s geological data (including the coal seam geology, the stratum development situation, the praedial-block feature, the petrographical compositions, the roof characteristics, information about other nearby wells, the gas output, water the output, and rainfall), the geological workers inferred that the gushing water in the stratum increased during that period, which increased the working level, indirectly indicating that the measurement results of the sensor well matched the practical geological conditions. This proves the error of the sensor from another perspective. Furthermore, it has been verified that the sensor was adaptable and stable during the nine-month period.

## 5. Cost Analysis and Comparison

The qualitative analysis of the cost of existing measurement methods is as follows:

① The cost of the sensor produced in this paper was low, but the installation cost was high due to the need for tubing during installation.
② The production cost of the buoy method was low, and the installation cost was lower as there was no need to unload the tubing during installation, while the accuracy was poor.
③ The echo sounding method in the wellhead and underground was required to install the test device due to the need to unload the tub during the installation, so the production cost and installation costs were relatively high.
④ The pressure-out method was less expensive to manufacture, but the installation requires that the tubing is unloaded, so the installation cost was higher.
⑤ Mathematical modeling calculations do not require the production of sensors or the unloading of tubing, but it needs to use a dedicated sensor to measure other parameters of field devices. Therefore, the cost is based on the type of measurement of the required parameters, but the installation cost is generally not high.

## 6. Conclusions

To meet the requirements for measuring the working level in a CBM wellbore annulus, a new sensor was designed. Using a bubble probe to estimate the density of the two-phase flow together with a pressure detection approach, this sensor could be used for the real-time measurement of the height. After the sensor was designed, the sensor was tested both in the lab and on site. The test results showed the following:

- The sensor was accurate to ±8%.
- The sensor could function well in practical conditions and remain stable over a long period.
- By analyzing the working principle of the designed sensor, we learnt that the working level could be calibrated only if the two-phase flow passed through the sensor probe. In other words, the sensor is directional and applicable to the two-phase flow moving in only one direction.
- The position of the sensor had an effect on measurement error. When the position was closer to the working level, the measurement was more accurate. If the working level fluctuated dramatically, it sometimes ended up below the sensor, which made the sensor unusable. Therefore, the position must be carefully determined by examining the surrounding geological conditions to ensure that the sensor is always below the working level.

## Figures and Tables

**Table 1:** Measurement index of working level sensor.

Parameters	Value
Measurement Range	0–1200 m
Error	±8%
Output Signal form	Figure Signal
Power Voltage	Direct Voltage 5 V
Applicable Medium	Gas–liquid Two-phase Flow
Sealability	0–4 MPa
Temperature	0–85 °C

**Table 2:** Automatic identification principles of two-phase flow patterns.

Patterns Time	0	*t*_1_	*t*_2_	*t*_3_
Bubble flow	Several bubbles		×	
Slug flow and churn flow	Only one bubble occurs		Liquid phase occurs	×
Annular flow and fine beam annular flow	Only one bubble occurs that contains a small amount of liquid phase			This bubble still exists and contains a small amount of liquid phase
Gas phase	Pure gas			
Liquid phase	Pure liquid			

**Table 3:** Relationship between the patterns and densities of the fluid.

Patterns	Density
Bubble flow	0.720
Slug flow or churn flow	0.561
Annular flow or fine beam annular flow	0.176
Liquid phase	1
Gas phase	0
